# Rolling Circle Amplification as an Efficient Analytical Tool for Rapid Detection of Contaminants in Aqueous Environments

**DOI:** 10.3390/bios11100352

**Published:** 2021-09-23

**Authors:** Kuankuan Zhang, Hua Zhang, Haorui Cao, Yu Jiang, Kang Mao, Zhugen Yang

**Affiliations:** 1 State Key Laboratory of Environmental Geochemistry, Institute of Geochemistry, Chinese Academy of Sciences, Guiyang 550081, China; zhangkuankuan@mail.gyig.ac.cn (K.Z.); caohaorui@mail.gyig.ac.cn (H.C.); jiangyu@mail.gyig.ac.cn (Y.J.); 2 University of Chinese Academy of Sciences, Beijing 100049, China; 3 Cranfield Water Science Institute, Cranfield University, Cranfield MK43 0AL, UK; Zhugen.Yang@cranfield.ac.uk

**Keywords:** rolling circle amplification, environmental monitoring, heavy metals, organic molecules, microorganisms

## Abstract

Environmental contaminants are a global concern, and an effective strategy for remediation is to develop a rapid, on-site, and affordable monitoring method. However, this remains challenging, especially with regard to the detection of various contaminants in complex water environments. The application of molecular methods has recently attracted increasing attention; for example, rolling circle amplification (RCA) is an isothermal enzymatic process in which a short nucleic acid primer is amplified to form a long single-stranded nucleic acid using a circular template and special nucleic acid polymerases. Furthermore, this approach can be further engineered into a device for point-of-need monitoring of environmental pollutants. In this paper, we describe the fundamental principles of RCA and the advantages and disadvantages of RCA assays. Then, we discuss the recently developed RCA-based tools for environmental analysis to determine various targets, including heavy metals, organic small molecules, nucleic acids, peptides, proteins, and even microorganisms in aqueous environments. Finally, we summarize the challenges and outline strategies for the advancement of this technique for application in contaminant monitoring.

## 1. Introduction

In recent years, the discharge of contaminants from industrial and agricultural activities and urban wastewater has caused serious contamination of the aqueous system, posing a great potential threat to human health and aquatic life. These contaminants can be divided into three categories: (i) inorganic chemical substances, (ii) organic pollutants and (iii) microorganisms. These substances can cause adverse effects on the environment [1,2,3,4], for example, the disruption of hormones and the endocrine system and the induction of cytotoxicity and/or genotoxicity and carcinogenesis [5,6]. The variable composition of pollutants and their location in aqueous environments over time have resulted in increasing focus on new technologies that use cheap and real-time strategies to monitor pollutants. Most of these strategies are based on laboratory platforms, such as inductively coupled plasma mass spectrometry (ICP-MS) for the detection of heavy metal ions, liquid chromatography-tandem mass spectrometry (LC-MS) for the detection of small organic chemicals or their metabolites, and polymerase chain reaction (PCR) for the detection of nucleic acids and genetic information, which require preprocessing and frequent data sampling, which means that they are both expensive and slow. These aspects highlight the need to develop a new strategy that is more sensitive, portable, and efficient for on-site detection of pollutants composed of multiple substances [7,8,9,10].

Recently, rolling circle amplification (RCA)-based analytical methods have received increasing attention in environmental monitoring. RCA is an uncomplicated and efficient isothermal enzymatic process using unique DNA and RNA polymerases to produce long single-stranded DNA (ssDNA) and RNA [11,12]. In RCA, the polymerase will spontaneously and continuously add nucleotides to the primers that bind to the circular template, generating long ssDNA with tandem repeats of tens to hundreds of orders of magnitude. Unlike PCR, which requires a thermal cycler and thermostable DNA polymerase. RCA can be in solution, on a solid support, or in a complex biological environment at a constant temperature (room temperature to 37 °C). The ability of RCA to grow a long DNA chain on a solid support or inside a cell from one molecular binding event enables the detection of targets at a single molecule level [13,14,15]. In addition, an RCA product comprising repeating cyclic sequences complementary to template DNA can be customized by template design. By designing the template, the customizable DNA product includes functional sequences, including aptamers, DNAzymes, spacer domains, and restriction endonuclease sites. Of course, by hybridizing the RCA product with a complementary nucleic acid linked to a functional part including biotin [16,17], fluorophores [18,19], antibodies [20], and nanoparticles [21,22,23,24], it is easy to synthesize a multifunctional material with a variety of properties, including biorecognition and biosensing. Collectively, the properties of high-efficiency isothermal amplification, single-molecule sensitivity, versatility of structure and composition, and multivalences make RCA a powerful tool in aqueous environments [25,26,27]. Currently, RCA has been extensively studied to develop sensitive methods for detecting DNA, RNA, DNA methylation, single nucleotide polymorphisms, small molecules, proteins, and cells. In addition to diagnosis, RCA has also been proven to be effective for cell-free cloning and sequencing [28,29], in situ genotyping and genome-wide analysis of cells and tissues [30,31,32,33,34]. Recently, RCA has received widespread attention for its use in the production of DNA nanostructures such as origami, nanoribbons, nanotubes, DNA nanoscaffolds, and DNA metamaterials for periodic nanocomponents [11,35,36,37,38]. Importantly, these materials have high prospects in a wide range of applications, including environmental monitoring, drug delivery, and in vivo imaging of manufacturing electronic circuits, including DNA-based materials.

In this article, we outline the basic engineering principles for implementing RCA design. Then, we discuss the progress of research in the last five years using RCA technology to analyse various pollutants in the water environment. Taking four kinds of common analytes in the environment as examples, including heavy metal ions, organic molecules, biological macromolecules, bacteria, and other microorganisms, the application of RCA for water environment monitoring is discussed. Among the analytes, biomacromolecules are divided into nucleic acids, lipids, peptides, and proteins. Finally, the contents of this paper are summarized, and the application prospects of RCA-based analytical methods in environmental monitoring are discussed.

## 2. Advantages and Disadvantages of the RCA Assay

### 2.1. Fundamentals of RCA

The RCA reaction typically requires four components: (1) DNA polymerase (e.g., Phi29 DNA polymerase), which includes an appropriate buffer; (2) a short nucleic acid primer; (3) a circular DNA template; and (4) deoxynucleotide triphosphate (dNTP) (monomer or structural unit of RCA product) [11,39,40,41]. In polymerases, Phi29 DNA polymerase is most commonly used because of its excellent capability and continuous strand displacement synthesis capability. Phi29 can handle topological constraints, four-way cross connections, and multiple circular DNA template complexes [39,42,43]. For RCA primers, both RNA and DNA (usually the “target” molecule to be detected) can achieve this goal. Indeed, the target DNA and RNA can be used to connect the first template mediated as a padlock probe (PLP) using RCA reaction circular template cyclizing [44,45]. The circular DNA template (usually 15–200 nucleotides (NT) in length) is a component that can be enzymatically or chemically synthesized through intramolecular phosphate and hydroxyl end groups. Most commonly, the template is a circular DNA template mediated by enzymatic ligation (e.g., T4 DNA ligase) or the use of a special DNA ligase enzyme with a template-free connection to a synthetic CircLigase [15,46]. By designing primers and circular templates, RCA product length, sequence, composition, structure and rigidity may be appropriately adjusted, thereby becoming a highly versatile RCA technique (summarized in Table 1).

### 2.2. Exponential RCA Amplification

**Figure 1:**
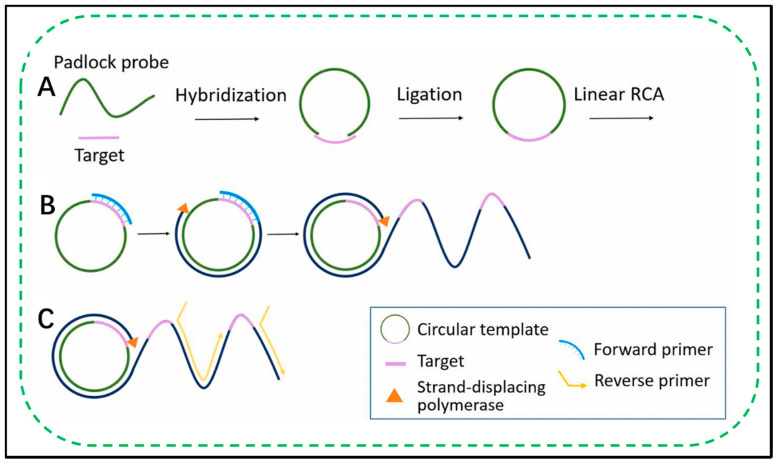
Schematics of RCA mechanisms. (**A**) Ligation RCA. (**B**) Linear RCA. (**C**) Hyperbranched RCA. Adapted with permission from ref. [50].

One of the powerful functions of RCA is the ability to design a circular template so that the signal generated by a single binding event is exponentially amplified [47,48,49]. Using a plurality of primers hybridizing to the same ring can lead to amplification of a plurality of events, thereby producing a plurality of RCA products [50,51] (Figure 1). The number of primers that one circular template can accommodate depends on the length of the primers and the circle. Another method for exponential amplification of RCA uses a so-called hyperbranched RCA (HRCA) (branched or amplification) method, in which the RCA product used as a template for the second and third groups is further expanded using primers [52,53,54,55]. Note that a primer can be integrated into the hyperbranched RCA method to increase the sensitivity, especially when the target is detected at low abundance [56]. Additionally, restriction enzyme digestion followed by enzymatic ligation template-mediated, linear RCA products may be converted to a variety of cyclic products [57,58]. A second set of primers may then be used to incorporate these new cyclic products for further amplification. This “circle to circle amplification” restriction digestion process, cyclization and amplification may be repeated for additional amplification. Finally, after hybridization with a second set of circles, the RCA product may be treated with nicking enzyme to generate a plurality of primers. The hybridized primer/circular template product obtained from the nickase reaction can be directly used for the next cycle of RCA amplification.

### 2.3. Detection of the RCA Product

A variety of signal reading technologies can be used to monitor and detect RCA processes and products. The most common RCA product analysis was carried out by gel electrophoresis. Furthermore, during RCA fluorophore dNTP coupling, fluorescent dye incorporated into the RCA product, bound by a fluorophore, or a complementary strand, can be easily observed using fluorescence-based techniques, including fluorescence spectroscopy, microscopy and flow cytometry [26,59,60]. Combining RCA with molecules such as modified AuNPs, quantum dots, or magnetic beads, it is easy to achieve visualization of RCA products [18,22,61,62]. For instance, RCA products can trigger the assembly of AuNPs for colorimetric and spectral visualization. RCA products can also be combined with magnetic beads to generate diffraction signals.

An electrochemical signal can also be generated by QD hybridization of the RCA products followed by dissolution to achieve high sensitivity [63]. Another method of detecting an electrochemical signal generated by an RCA product comprises inserting the DNA into an organic molecule (e.g., methylene blue), which is inserted into the RCA product [64]. Molecular beacons [65,66] and DNA-intercalating dyes (e.g., SYBR green [67,68]) have also been widely used to detect RCA products, which is also important since real-time monitoring of the reaction in the absence of RCA DNA products results in minimal background fluorescence. In addition to the fluorescence signal, there are some intercalating dyes, such as 3,3-diethylthiadicarbocyanine iodide (DiSC2(5)), that, when combined with the duplex between DNA and peptide nucleic acid (PNA), can be used to produce a colour change from blue to purple [69]. Then, the colour change signal can achieve the goals of the naked eye for detection, which is particularly suitable for real-time diagnostic applications. Furthermore, HRP (horseradish peroxidase) was immobilized on RCA products through biotin modification of DNA to realize visual detection based on colorimetry [70]. Interestingly, the DNAzyme sequence that mimics HRP can catalyse the oxidation of 2,20-azidobis (3-ethylbenzothiazolin-6-sulfonic acid) and generate a blue-green colorimetric signal. It can also be incorporated into RCA products [71]. This “dual-amplification system” (i.e., the RCA and has multiple converting enzyme DNA) enables real-time supersensitive colorimetric detection of target molecules. Finally, the RCA product can be detected by bioluminescence. In this case, the RCA reaction generates a large amount of pyrophosphate that can be used as an adenyl transferase substrate to produce ATP. Then, firefly luciferase ATP acts as a cofactor to produce a bioluminescent signal [72,73].

RCA can not only achieve signal amplification of target nucleic acids through amplification but also has flexible and diverse visualization methods; therefore, it has great potential for application in nucleic acid detection. The advantages of RCA include the following: (1) high sensitivity: RCA has strong amplification ability, the efficiency of exponential RCA can reach 10^9^ fold, and it has the potential to detect single copies; (2) high sequence specificity: it can distinguish single nucleotide polymorphisms; (3) the amplified product can be directly used for sequencing after phosphorylation treatment; (4) high throughput: RCA can form a closed circular sequence on the target, ensuring that the signal generated by RCA is concentrated at one point, thereby achieving in situ amplification and slide amplification. However, there are still some shortcomings in the development of the RCA method: (1) the padlock probe is often close to 100 bp, and therefore, the synthesis cost is relatively high; (2) background interference is a problem during signal detection.

## 3. RCA Assay for the Detection of Targets in Aqueous Environments

Over the years, a number of RCA assays have been developed for the sensitive and specific detection of various targets, including heavy metals, organic small molecules, nucleic acids, peptides and proteins, and microorganisms in aqueous environments (listed in Table 2).

### 3.1. RCA Assay for Heavy Metal Ions

The basic definition of heavy metal elements refers to any metal element that has a relatively high density and is poisonous or toxic even at low concentrations, such as lead (Pb), cadmium (Cd), mercury (Hg), chromium (Cr), and arsenic (As). As the chemical properties of arsenic are similar to those of heavy metals, arsenic is also grouped with heavy metals [101]. Heavy metal pollution has gradually developed as an environmental problem affecting human health in many countries [102,103,104]. Therefore, the development of sensitive and selective heavy metal ion detection methods is imperative to preserve the environment and protect human health.

Traditional methods developed for heavy metal ion detection include chromatographic, spectroscopic, and electrochemical methods. These techniques have the advantages of high accuracy and sensitivity; however, expensive and complicated instruments, complicated sample preparation, and well-trained operators are all indispensable, which means they cannot meet the requirements of portability and ease of use. Recently, novel RCA-based methods have shown great potential in heavy metal ion detection in aqueous environments due to their advantages of low cost and easy operation. Thus, the following sections review some of the research efforts in this area in recent years [105].

#### 3.1.1. Mercury (Hg)

In recent decades, mercury pollution has been commonly found in water, food, cosmetics, the atmosphere, and human health and poses a serious threat to the economy [106]. Contaminants of mercury exist in different forms in nature, such as elemental mercury, HgCl_2_, Hg_2_Cl_2_, methyl mercury (CH_3_Hg), and Hg(NH_2_)Cl. These molecules can be ingested, absorbed into the body (through the skin) and inhaled and accumulate in vital organs and tissues, leading to organ dysfunction and irreversible damage to the nervous system. Therefore, the World Health Organization (WHO) has determined the maximum allowable mercury content in different samples to control the harm caused by mercury. Fast, simple, and cost-effective development of in situ testing methods will facilitate the management of heavy metal pollution and mitigation [107].

**Figure 2:**
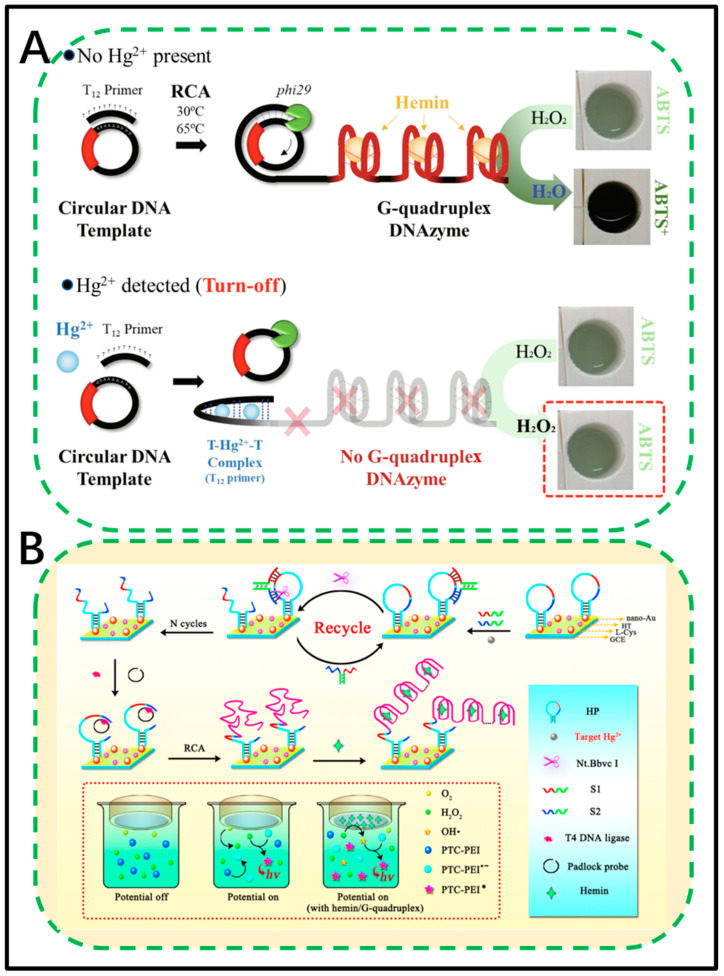
RCA assay for Hg detection. Adapted with permission from ref. [27,79]. (**A**) Colorimetric change of the detection chip when there are no mercury ions in the sample and when mercury ions are present. (**B**) Signal opening sensor based on novel covalently crosslinked perylene derivative (PTC-PEI) system design.

The colorimetric detection that converts density information into colour changes can be directly interpreted by the naked eye terminal. Due to the low cost, portability, and ease of operation of the colorimetric method, it has been widely and routinely used for the detection of various targets, such as DNA [108,109], proteins [110], cells [111,112,113], and heavy metal ions [114]. The combination of RCA and colorimetric assays has also been used to analyse heavy metals in aqueous environments. Wang et al. developed an RCA detection method based on a signal enhancement Hg colorimetric aptasensor [78]. As Hg^2+^ poses a serious threat to public health and food safety, the technology for sensitive detection of Hg^2+^ is constantly innovating. In a previous study, Lim et al. constructed an instant detection chip for the colorimetric detection of inorganic Hg^2+^ based on microfluidics that was portable and easy to operate [79]. Manufactured by a three-dimensional printing technique, a disposable chip comprising DNAzyme RCA was generated. A colour change caused by the enzymatic reaction between DNAzymes and the peroxidase substrate 2,2′-azino-bis(3-ethylbenzthiazoline-6-sulfonic acid) (ABTS) was measured using a portable spectrophotometer (Figure 2A). In the “turn-off” type RCA reaction, the interaction of thymine with Hg^2+^ prevents the annealing of the T-rich primer that initiates the RCA reaction. Therefore, depending on the Hg^2+^ concentration, the number of amplified DNases that cause colour changes is reduced. The colorimetric signal is enhanced by amplifying double-repeat DNAzymes from a circular DNA template. The chip detects Hg^2+^ in tap water samples with a high sensitivity of 3.6 μg L^−1^. Compared with conventional analytical instruments, it has higher selectivity, precision, and reproducibility. This low-cost, easy-to-use platform can reduce the risk of accidental poisoning by Hg^2+^.

Similarly, Wu et al. recently fabricated a colorimetric aptamer sensor based on RCA to detect Hg^2+^ which possesses an even lower detection limit [78]. First, the aptamer hybridized with its complementary strand (cDNA_1_) is fixed on the microtiter plate, and the complementary strand (cDNA_1_) is connected to the primer at the same time to trigger the RCA reaction of the circular template. A successful RCA process will result in the formation of long ssDNA strands on the microtiter plate, resulting in DNA fragments that hybridize with cDNA_2_ from many organisms. The avidin/biotin binding between avi-HRP and bio-cDNA_2_ increases the amount of labelled HRP. By adding TMB-H_2_O_2_, HRP catalyses the reaction and generates a light signal. When there is a target, the situation will be completely different. Hg^2+^ preferentially binds to the aptamer to form a strong and stable T-Hg^2+^-T complex, resulting in the release of the HRP cDNA_1_ cluster. Therefore, the optical signal is reduced. The results show that the limit of detection (LOD) was 1.6 nM, with excellent specificity. Compared with the detection signal of the RCA-free system, the detection signal can be increased up to 18 times.

Among different detection methods, fluorescent strategies have unique properties, such as easy installation, suitable signal transduction, a wide linear range and quick response. For instance, Chen et al. reported a highly sensitive Hg^2+^ fluorescent sensor based on hyperbranched RCA [74], with a detection limit of 0.14 pM. More recently, Zhao et al. established a method that used trifunctional molecular beacon-mediated quadratic amplification for the highly sensitive and rapid detection of Hg^2+^ with a tunable dynamic range [76]. Due to its moderate sensitivity and limited dynamic range, it is challenging to analyse targets with low abundance or multiple orders of magnitude changes in concentration. Here, the authors introduced a homogeneous and fast quadratic polynomial amplification strategy by rationally designing three functional molecular beacons. This strategy not only acts as a reporter but also acts as a coupled two-stage amplification module without adding any bridges of reaction components or processes. The Hg^2+^ assay as an example and achieved high sensitivity with an LOD of 200 pM within 30 min. To create an adjustable dynamic range, isomorphisms are used to regulate target-specific binding. When the number of metal binding sites changes from one to three, the useful dynamic range (spanning 50-, 25-, and 10-fold) is used to program the signal response accordingly. In addition, the applicability of this method in river water samples has been successfully verified, and it has good recovery and reproducibility, indicating that it has great practicability in complex actual samples.

Electrochemical response signals are fast, inexpensive, and can be miniaturized for use with other portable devices, which enables the use of very few samples by nontechnical personnel to measure a target on the spot; thus, electrochemical methods have attracted increasing attention. Zhao et al. developed a novel perylene derivative with electrochemiluminescence (ECL) and applied it to Hg^2+^ detection based on a dual amplification strategy [27]. The cathodic ECL of a new covalently cross-linked perylene derivative (PTC-PEI) composed of polyethyleneimine (PEI) and perylene tetracarboxylic acid (PTCA) in an aqueous system was first studied (Figure 2B). Promising novel materials with ECL in PTC-PEI exhibit excellent physical and chemical stability and high ECL intensity, presenting an alternative way to construct an ECL sensor with improved sensitivity. Thus, this sensor was applied to construct a dual amplified ‘‘signal-on’’ Hg^2+^ sensor by employing nicking endonuclease (NEase)-assisted target recycling and RCA for semaphore amplification. Herein, the process is produced by RCA of a long G-rich sequence to capture large amounts of haem on the electrode surface, and then a significant amplification of ECL signals by a PTC-PEI is obtained. This sensor platform showed a detection limit as low as 33 fM with a wide linear range from 0.1 pM to 0.1 μΜ. Based on the dual-signal amplification strategy, the designed sensor was successfully used to directly detect real water samples from lakes using the standard addition method.

Thymine-Hg^2+^-thymine (T-Hg^2+^-T) induces DNA strand replacement and realizes the specific recognition of Hg^2+^. It is a common strategy for Hg^2+^ detection in environmental samples. Lv et al. developed an ultrasensitive electrochemical measurement method for Hg^2+^ using an efficient target conversion method [77]. First, AuNPs were uniformly coated on polystyrene magnetic microspheres as a magnetic separator, and then ssDNA D1 (rich in thymine) and S1/D2 DNA duplexes (rich in guanine S1) were used as markers. When Hg^2+^ and long ssDNA D3 (rich in thymine at the 5′ end) are present in the tested sample, a stable T-Hg^2+^-T structure between D2 and D3 is immediately formed, and S1 is changed from the S1/D2 DNA duplex, thus realizing the transformation of S1. At this time, the target Hg^2+^ is combined with the output S1. Therefore, the total amount of output S1 is proportional to the amount of input Hg^2+^. After that, the output S1 will be used as a primer to start an RCA reaction to obtain long guanine-rich ssDNA, thus achieving further hybridization with the DNA captured on the electrode surface. Eventually, methylene blue, as an electron mediator, will interact with the ssDNA polymer through electrostatic binding to generate a detection signal. The electrochemical biosensor based on RCA has a wide linear range, good accuracy, and excellent recovery rate. These stable properties are very suitable for water sample detection. It has strong competitiveness and application in detecting Hg^2+^ in the environment.

#### 3.1.2. Lead (Pb)

**Figure 3:**
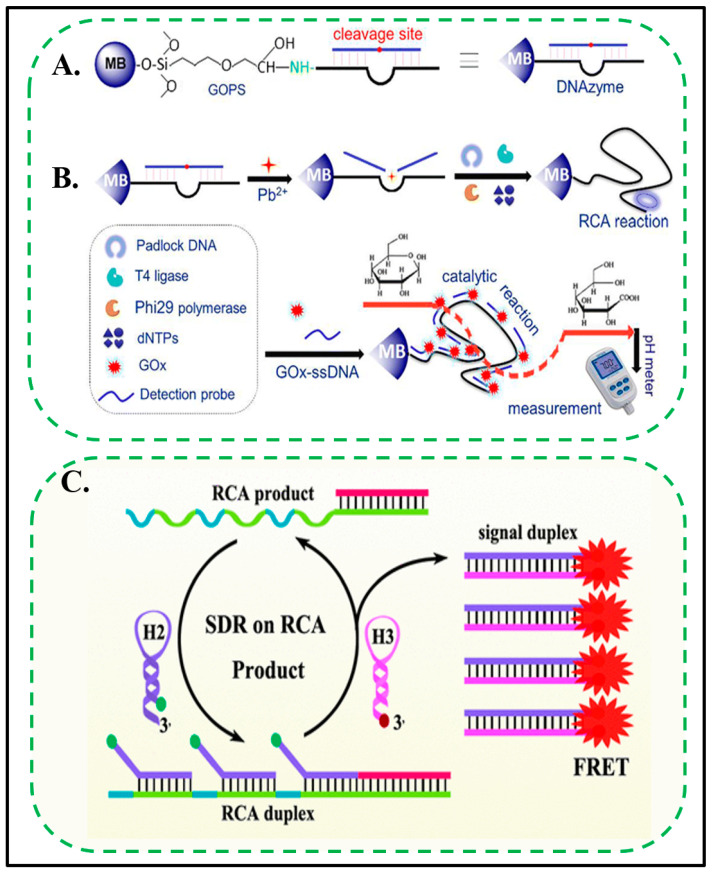
RCA assay for Pb^2+^ detection. Adapted with permission from ref. [60,81]. (**A**,**B**) Illustration of metal-ion-induced DNAzyme on magnetic beads (MB) for the detection of Pb^2+^ with rolling circle amplification (RCA) on a handheld pH metre; (**C**) Multisite-strand-displacement-reaction (SDR) signal-amplification strategy.

Lead ions (Pb^2+^) are highly toxic heavy metal pollutants that are widespread in the water environment. Since lead ions bioaccumulate and have nonbiodegradable properties, even at low concentrations, lead ions may also cause nervous, reproductive, cardiovascular, and other developmental disorders [115]. Figure 3C demonstrates an ultrasensitive fluorescent assay based on an RCA-assisted multisite-strand-displacement-reaction (SDR) signal-amplification strategy [60]. The proposed strategy is not only to achieve recycling targets but also to introduce RCA by the release of the DNA enzyme. Most importantly, the RCA product is used as an initiator to provide a plurality of SDR sites, which can replace the duplex signal RCA product to effectively prevent the self-quenching probe assembly RCA product signal. Therefore, the amplification efficiency and sensitivity can be significantly improved. Using this strategy for intracellular Pb^2+^ detection, a detection limit as low as 0.03 nM and a wide linear range from 0.1 pM to 0.1 μΜ were obtained. In addition, the proposed strategy can be extended to determine other goals and provide a new approach for environmental analysis.

Liu et al. developed a rapid and sensitive method for Pb^2+^ detection based on a cationic conjugated polymer and an aptamer [116]. By selecting a more specific aptamer probe, the probe for Pb^2+^ recognition and combination is a single-stranded oligonucleotide labelled with fluorescein. Upon combining with Pb^2+^ with high specificity, the random coiled probe changed to a G-quadruplex with a higher charge density, which enhanced the electrostatic interactions between the oligonucleotide and the cationic conjugated polymer; thus, the two fluorophores were in close proximity, leading to a significantly increased fluorescence resonance energy transfer (FRET) signal. However, other nontarget metal ions produced much lower FRET signals because they could not combine with the probe and thus quenched the fluorescence of the conjugated polymer and fluorescein. This method was rapid, highly specific, and sensitive, and common metal ions did not influence the detection of Pb^2+^. This FRET-based method, whose LOD was lower than the national standard for drinking water quality, provides a new simple, rapid, and efficient method for the detection of Pb^2+^ in various sources of water.

Using the device integration technique, Tang et al. designed a metal-ion-induced DNAzyme on magnetic beads for Pb^2+^ detection by using RCA, glucose oxidase, and a readout of pH changes [81]. As shown in Figure 3A, the work reported a method of measuring Pb^2+^ ions in environmental samples. A Pb^2+^-specific DNAzyme immobilized on magnetic beads was coupled to RCA and a pH-metre-based readout. The addition of Pb^2+^ ions induced partial cleavage of the DNA enzymes on the magnetic beads. The single-stranded DNA retained on the magnetic beads was used as a primer. With the help of a circular DNA template, polymerase and dNTPs trigger the RCA reaction. This results in the formation of many oligonucleotide repeats on the magnetic beads. Subsequently, these repetitive sequences are hybridized with glucose oxidase-labelled single-stranded DNA (GOx-ssDNA) to form a long coenzyme containing tens to hundreds of GOx-ssDNA tandem repeats (Figure 3B). The linked GOx molecules oxidize glucose, which is accompanied by a decrease in local pH. This method has good reproducibility, high specificity, and acceptable accuracy. It is used to analyse spiked water samples, and the results are superior to those obtained by ICP-MS.

Rapid, portable, and efficient Pb^2+^ detection is important for monitoring environmental toxicity and evaluating human health. Lu et al. demonstrated a DNAzyme assay coupled with effective magnetic separation and RCA for the detection of Pb^2+^ with a smartphone camera [80]. In this work, a simple and low-cost homogenous fluorescence DNAzyme assay was developed for Pb^2+^ determination based on Pb^2+^-dependent cleavage and RCA. A DNAzyme and its substrate form a double-stranded hybrid in solution, which can completely react with Pb^2+^ in the water phase. Then, DNAzyme/substrate hybrid and unreacted substrate portions with chain cleavage of the biotin-labelled biotin-streptavidin interaction avidin magnetic beads are captured and removed from the reaction solution. The rest of the substrate chain remains in solution and then acts as a primer and triggers RCA. The concentration of cleaved substrate strand Pb^2+^ concentration related, and the biotin-streptavidin-biotin separation was effective to minimize non-specific amplification. Using a smartphone camera, the fluorescence intensity was recorded and quantified after 30–90 min of amplification so that this method could be carried out with the least amount of equipment. Under the best conditions, the dynamic range is 1–100 nM, and this method has been successfully used for the detection of Pb^2+^ in spiked lake water [80].

Tsekenis et al. developed heavy metal ion detection using a capacitive micromechanical biosensor array for environmental monitoring [117]. In this work, the fabrication and evaluation of a DNAzyme-functionalized capacitive micromechanical sensor array for the detection of lead ions is proposed. In the presence of Pb^2+^, the enzyme may catalyse DNA chain cleavage of the substrate DNA strand with ribonucleotide bases to dissociate the complex into three segments. The DNAzyme strand is laser printed and fixed on the sensor surface and hybridizes with the substrate strand. When self-cleavage occurs, the surface stress will change, which is then recorded as a change in device capacitance. The sensor can detect 10 μM Pb^2+^, and in the reverse process, it proves the rehybridization of the immobilized catalytic chain and the substrate chain. The reaction is verified by labelling the catalytic chain with a fluorescent molecule, while the substrate chain is labelled with a quencher.

#### 3.1.3. Other Ions

In addition to Hg^2+^ and Pb^2+^, other ions have also been reported, such as uranyl ions (UO_2_^2+^). Chen et al. developed a visual detection method for ultratrace levels of uranyl ions using magnetic bead-based DNAzyme recognition in combination with RCA [82]. The authors describe a colorimetric method for the determination of ultratrace levels of uranyl ion in beverages and milk. The employment of DNAzyme-functionalized magnetic beads facilitates the separation and collection of the analyte from the sample matrix. The RCA strategy achieves an effect with a ratio of one UO_2_^2+^ to massive amounts of HRP, which strongly improves the sensitivity. The visual detection limit is much lower than the maximum allowable level of UO_2_^2+^ in drinking water as defined by the USA Environmental Protection Agency, which indicates that the method meets the requirements for simple, rapid, and on-site detection of ultratrace UO_2_^2+^ in real samples.

### 3.2. RCA Assay for Organic Small Molecules

**Figure 4:**
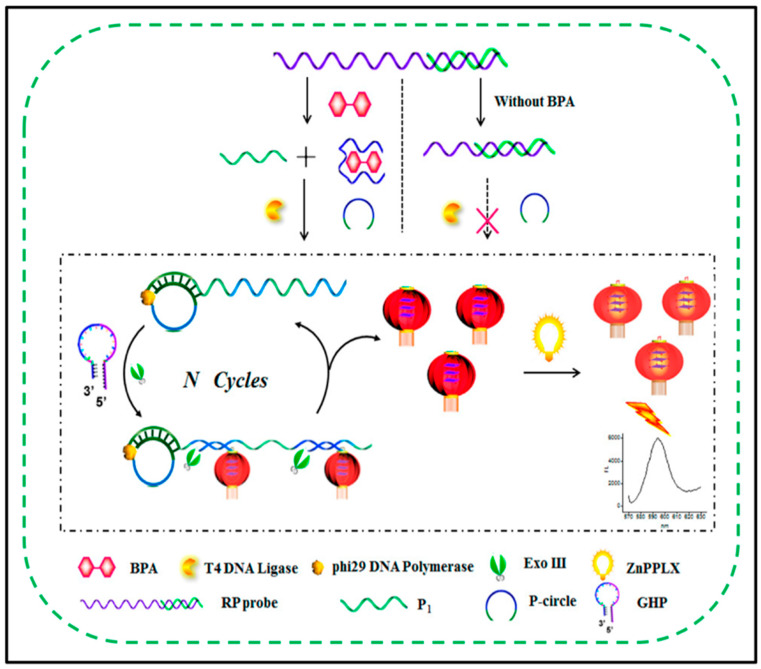
A label-free and sensitive fluorescent qualitative assay for bisphenol A based on RCA/exonuclease III combined cascade amplification. Adapted with permission from ref. [83].

Organic pollutants can come from natural or anthropogenic sources, and industrial, agricultural, and domestic wastewater can be found in a wide range of these pollutants. Among various organic pollutants, bisphenol A (BPA) is a typical substance that has caused widespread concern. As the scientific understanding of bisphenol A continues to deepen, it is especially discovered that it can cause disorder and damage to the normal physiological processes of the human body. Currently, major cities have strengthened the detection and supervision of bisphenol A (BPA) in terms of food safety and drinking water safety. However, the detection of BPA relies on precision and expensive machines, such as HPLC-ICP-MS. These methods require tedious operations and long analysis times. To reduce the analysis cost and simplify the operation, researchers have developed an RCA method to detect BPA. For instance, Xia et al. creatively constructed a label-free aptamer fluorescence sensing platform based on the RCA/Exo III (Exo III) combined cascade amplification strategy, which has high selectivity and high sensitivity for BPA detection [83] (Figure 4). The first step is to design a BPA-resistant aptamer and a DNA double-stranded probe (RP) for the trigger sequence for BPA recognition and signal amplification; next, when BPA appears, it will trigger the probe to be released. On this basis, the initial amplification reaction of RCA was started. When an increasing number of RCA products appear, the RCA products will trigger a second amplification assisted by Exo III with the help of hairpin probes. To date, many G quadruplexes will be enriched in lantern-like structures. Finally, by irradiating the G-quadruple lantern with zinc(II)-protoporphyrin IX (ZnPPIX), an enhanced fluorescence signal is generated. In the above process, RCA acts as the primary amplification, and the secondary Exo III mediates the secondary amplification. This cascade amplification gives the detection platform excellent sensitivity, and the detection limit is 5.4 × 10^−17^ M. The strong specificity of the anti-BPA aptamer guarantees the specificity of the platform. This kind of unlabelled fluorescent signal probe avoids the tedious labelling process, greatly reduces the design operation, and at the same time makes the cost lower. In the end, the author also carried out the measurement of real samples of the environment, and the results were reliable, demonstrating the potential application value of this method in the field of environmental detection.

### 3.3. RCA Assay for Nucleic Acids

MicroRNAs (miRNAs) are evolutionarily conserved, ~18–24-nucleotide-long noncoding RNAs that play a significant role in the control of human gene expression by posttranscriptional gene regulation or silencing. Furthermore, the abnormal expression of a single miRNA can regulate the activity of multiple genes. Previous studies have shown that changes in miRNA expression may lead to a variety of human diseases and disorders, such as cancer, cardiovascular disease, autoimmune disease, neurodegenerative disease, and liver and inflammatory diseases [118,119]. miRNAs are very stable in human peripheral blood circulation and are widely present in other body tissues and fluids, such as urine, saliva, milk, and cerebrospinal fluid. These characteristics indicate that miRNAs are potential biomarkers for diagnostic purposes. miRNAs are related to the occurrence and development of diseases and are pathologically specific; therefore, altered miRNA expression has been used for early detection and diagnosis, classification, prognosis, and predictive diagnosis [120,121,122,123,124].

**Figure 5:**
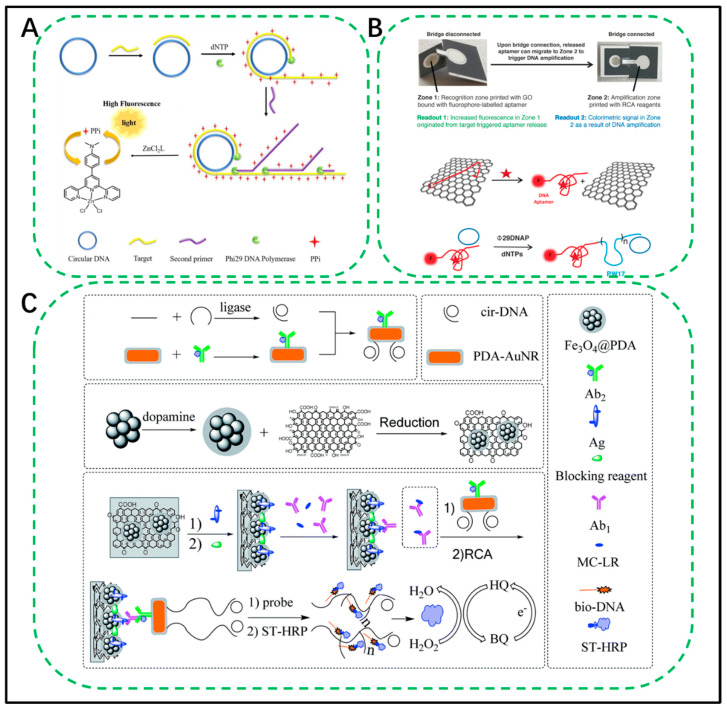
RCA assay for nucleic acid detection. Adapted with permission from ref. [84,88,89]. (**A**) Label-free miRNA sensor based on zinc terpyridine complex; (**B**) design of detachable paper-based biosensor with dual signal output; (**C**) construction of a dual-signal amplification immunosensing platform based on magnetic graphene. MC-LR: microcystin-leucine-arginine, PDA: polydopamine, Ab1: antigen (Ag) and antibody (primary antibody), ST-HR: streptavidin-HRP.

Ma et al. developed a fast, sensitive, and highly specific label-free fluorescent quantitative biosensor for miRNA through the branched-chain RCA (BRCA) reaction [84] (Figure 5A). The target miRNA acts as a primer and can hybridize specifically to the circular DNA template. Then, RCA is initiated by Phi29 DNA polymerase, and a reverse primer complementary to the RCA product is introduced during this process to achieve isothermal BRCA. While consuming a large amount of dNTPs, it produces the same number of pyrophosphate (PPi) molecules. In this study, a simple and cheaply synthesized pyridine-based Zn(II) complex was used as a fluorescent probe for the selective detection of PPi through dNTPs. In this way, the PPi generated during the isothermal amplification process is effectively chelated to the pyridine-Zn(II) complex to form a highly fluorescent complex, pyridine-Zn(II)-PPi, whose fluorescence intensity is only comparable to the original target. The concentration of miRNA is closely related. This strategy not only achieves isothermal amplification but also allows direct monitoring of DNA polymerization byproducts. For the nonlabelled fluorescence detection of miRNA, PPi greatly simplifies the sensor procedure. This noncumbersome sensor provides a sensitive and easy-to-use platform for miRNA quantification. Significantly promote the career of miRNA as a biomarker in drug discovery, clinical diagnosis, and life science research.

Zhou et al. designed a new high-throughput method to analyse the methylation pattern of individual DNA molecules [125]. High-throughput assays for methylation pattern analysis of individual DNA clones are important for research on tumour initiation, progression and transfer and chemotherapy. In this study, a new method was developed for methylation pattern analysis based on HRCA cloning and microarray technology. A library of DNA fragments with different methylation statuses was amplified from bisulfite-modified genomic DNA using PCR, and circular PCR products were then formed by ligation with a linker. HRCA was performed on streptavidin-coated beads in water-in-oil microemulsions, where the circular products were used as templates with one of the primers immobilized on the beads. Finally, the beads were immobilized on glass slides using polyacrylamide gel and hybridized with specific probes to identify the multiple C g site methylation status of each clone. This method was applied successfully to each clone methylation pattern analysis of the P16 gene promoter in 10 stomach tumour samples and 10 corresponding normal samples. The experiments showed that the method could measure the methylation pattern of each DNA clone with high sensitivity simply by counting the methylation clones.

Xu et al. developed an RCA integrated detection platform that can be used for multiple miRNA quantification by preparing a new type of porous hydrogel-encapsulated photonic crystal (PhC) barcode [85]. The development of a highly sensitive platform for the detection of multiple circulating miRNAs is very important for clinical diagnosis. The porous hydrogel shell and the hydrophilic protein scaffold are connected to each other to form an opal reverse structure in which the PhC barcode is coated. Opal anti-structure can provide homogeneous water around miRNA target reaction and RCA. The encapsulated PhC core of the barcode can provide stable diffraction peaks to encode different miRNAs and their RCAs during the detection process. In this way, the advantages of the PhC barcode and RCA are integrated. Experiments have proven that this technology shows acceptable accuracy and reproducibility for rapid quantification of low-abundance miRNA (20 fM). Therefore, the proposed porous hydrogel-encapsulated PhC barcode provides a new platform for multiple quantification of low-abundance targets in practical applications.

Xu et al. produced a sensitive nucleic acid detection platform based on superhydrophobic micropores [86]. The micropores are located on the superhydrophobic substrate. Due to the difference in wettability, ultratrace DNA molecules are enriched, which realizes the concentration of the chip, and then the fluorescence signal is amplified, which improves the detection sensitivity. Using the biosensing interface of ultrawet materials to detect ultratrace DNA through concentration has opened up a simple and cost-effective new way of thinking.

Based on the high dark phase contrast of vapour condensation, Zhang et al. developed a label-free smart device that can detect diseases related to gene mutation sites in real time [86]. A Peltier cooler and a mini PC board for image processing are the core components of the device. The workflow, in short, uses the heat of the hot end of the Peltier cooler to evaporate the fluid in the copper cavity, and then the vapour condenses on the surface of the microarray chip placed on the cold end of the cooler and further characterizes the vapour condensation relative to the microarray. The high dark phase contrast of the analytes on the chip. Used in conjunction with RCA, the device can see the change from reduced hydrophilicity to hydrophilicity caused by gene capture and DNA amplification. Analysis of lung cancer gene point mutations proved the high selectivity and multiple analysis capabilities of this inexpensive device.

### 3.4. RCA Assay for Peptides and Proteins

Figure 5C shows an RCA signal-enhanced immunosensor for ultrasensitive microcystin-LR (MC-LR) detection based on magnetic graphene-functionalized electrodes [88]. This novel competitive immunosensor promotes the development of the MC-LR detection field. Magnetic graphene is synthesized, characterized, and used as a substrate. Due to its large surface area and easy separation, the antigen can be immobilized on the electrode surface. In addition, gold nanorods modified with polydopamine are modified and functionalized with secondary antibodies and circular DNA templates. Through the function of RCA, the DNA template can be replicated to generate a large number of repetitive DNA sequences. At this time, the detection probe will hybridize with the repetitive sequence; therefore, the signal has been significantly improved. Under optimal parameter conditions, the immunosensor proposed by the team has a good linear relationship between the current response in the range of 0.01–50 μg L^−1^ and the target concentration, and the detection limit is 7.0 × 10^−3^ μg L^−1^. Similarly, the immunosensor has also been proven to be highly specific, and its reusability and stability are commendable. Most importantly, the proposed biosensor is used to detect MC-LR in real water samples and has a good recovery rate, indicating its application prospects in actual environmental monitoring. Hui et al. reported a paper-based multifunctional bio/nanomaterial printed sensor platform, as shown in Figure 5B [89]. The platform is divided into two reaction zones and a connecting bridge. Molecular recognition and signal amplification are realized by printing multifunctional biological/nanomaterials. When the targeted analyte appears in the first area, the fluorescently labelled nucleic acid aptamer will automatically desorb from the printed graphene oxide, thereby quickly generating an initial fluorescent signal. Then, the released aptamer flows with the wave to the second area, where it reacts with the printed reagent to initiate RCA, thereby generating DNA amplicons containing DNAzyme mimicking peroxidase, thereby generating colorimetric readings. No equipment or smartphone is required to interpret the reading. To verify the specificity and sensitivity of the sensor, the author used an adenosine triphosphate RNA aptamer (a bacterial marker) and glutamate dehydrogenase DNA aptamer for verification and successfully passed the test. Moreover, when the target is added to serum or stool samples, it can still be detected, proving the potential of this method in testing clinical samples.

### 3.5. RCA Assay for Microorganisms

Widespread waterborne microbial diseases have caused significant mortality and morbidity worldwide [126]. Therefore, the detection of these microorganisms or pathogens is very important. Conventional microbial pathogen detection methods require the use of artificial culture media or microscopic methods. These methods have many technical limitations, such as low detection accuracy, low sensitivity, complex sample processing and time consumption [127,128,129]. For instance, coliform assays are traditionally used to assess coliform bacteria in environmental samples but not to monitor the overall microorganism content.

Due to the abovementioned shortcomings of conventional methods, the development of technologies for more effective discovery of trace pathogens in dark water has received considerable attention. Recently, in the field, it has been applied in several molecular methods, such as PCR, enzyme-linked immunosorbent assay (ELISA) [130,131], and fluorescence in situ hybridization (FISH) [132], and applying policy-based biosensors to detect microorganisms in water and wastewater has become a very popular field of study [133]. The use of biosensors can identify microbial contamination in real time, while using traditional techniques, it takes several days to obtain results.

**Figure 6:**
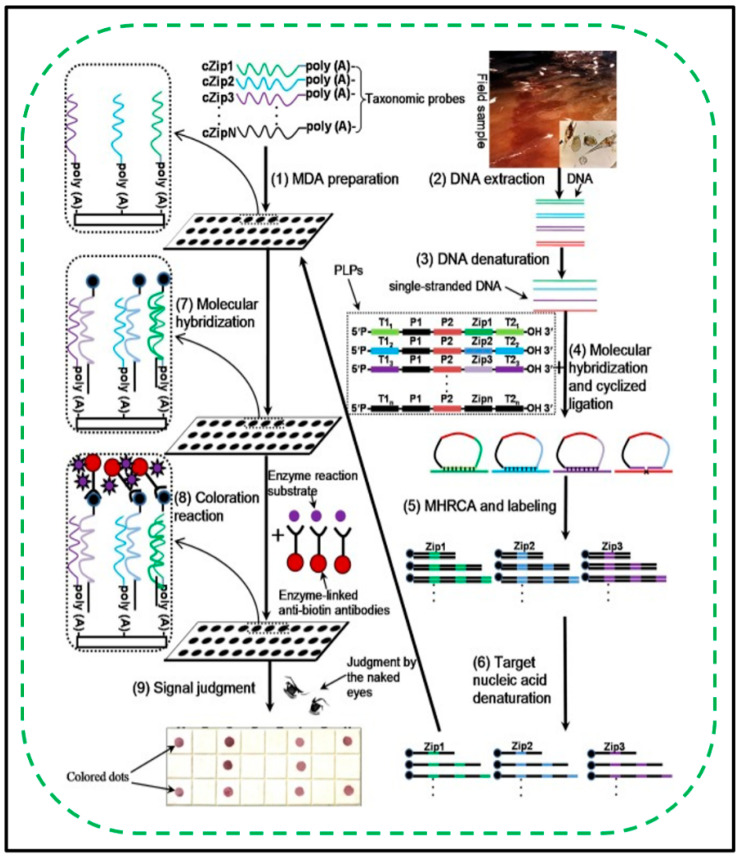
RCA assay for microorganism detection. Adapted with permission from ref. [92].

Harmful algal blooms (HABs) of toxic microalgae have received much attention worldwide because their existence has always been a threat to marine ecosystems, fisheries, and human health. The scientific community has also been developing a monitoring system that can effectively and accurately identify pathogenic algae and monitor the quality of seawater. Unfortunately, the traditional methods based on laboratory precision microscopes are too complicated and time-consuming [134,135]. Taking the coast of China as the research area, part of the large subunit rDNA (D1-D2) sequences of eight common toxic and harmful algae in the research area were cloned, and then a specific PLP consisting of universal primer binding sites and ZIP sequences was designed. Then, a sorting probe DNA array complementary to the ZIP sequence on the nylon membrane was prepared. The amplified product is labelled with biotin produced by multiple HRCAs (MHRCAs). After heat denaturation, the MHRCA product will hybridize with the DNA array, and then spot colouring will appear. As shown in Figure 6, an MHRCA-based membrane DNA array assay (MHBMDAA) for the detection of toxic microalgae has been developed [92]. The specificity of MHBMDAA was confirmed by the double cross-reactivity test of PLP and taxonomic probes. The detection range of the MHBMDAA method in simulated samples can reach 0.1 to 1000 cell mL^−1^, and its sensitivity is 10 times higher than that of the multiplex PCR membrane DNA array. The validity and reliability of MHBMDAA have also been verified by natural samples from the East China Sea. The results show that MHBMDAA is a sensitive and reliable detection tool for the early warning system of toxic microalgae.

*Karenia mikimotoi* (*K. mikimotoi*) is globally distributed, toxic, and harmful, and it easily forms water blooms. It is similar to a single spark, which explodes frequently in the global seas. To avoid endangering seafood and human health, fast, accurate, and sensitive on-site monitoring of this harmful algae is needed. Zhang et al. reported the comparative detection of *Carrenella triloba* through exponential RCA (E-RCA) and double-linked E-RCA and compared their sensitivity with traditional PCR methods [90]. Part of the large subunit rDNA (D1-D2) of *K. mikimotoi* was amplified, cloned, and then sequenced. After the obtained sequence was used for specific region comparison analysis, PLP and primers of E-RCA and dlE-RCA were designed. Through the simulation, the parameters of the E-RCA and dlE-RCA systems are optimized. The specificity test showed that both E-RCA and dlE-RCA are specific to *K. mikimotoi* bacteria. The sensitivity comparison shows that the sensitivity of E-RCA is 10 times higher than that of PCR, while the sensitivity of dlE-RCA is equivalent to that of PCR. Tests on simulated field samples show that the detection limits of the developed E-RCA and dlE-RCA methods are 1 and 10 cells, respectively. By visually observing the colour reaction and adding fluorescent SYBR green I dye to the reaction tube, it can be confirmed that E-RCA and dlE-RCA are positive. Compared with E-RCA, dlE-RCA can avoid the self-cyclization of PLP. The developed E-RCA and dlE-RCA methods are also very effective for field samples with a target cell density in the range of 10–1000 cells mL^−1^. These results indicate that the established E-RCA and dlE-RCA detection protocols show expectations for future field applications of *K. mikimotoi* monitoring.

Zhang et al. established a method combining isothermal amplification technology and a rapid analysis method for the rapid detection of *K. mikimotoi* on site [90]. In short, it consists of two parts: HRCA isothermal amplification based on targeted nucleic acids and lateral flow dipstick (LFD) for detecting nucleic acid amplification products, namely, the HRCA-LFD sensor analysis platform, which relies on targeted DNA template PLP and LFD probes targeting PLP to detect *K. mikimotoi*. The core point is the sequenced endogenous *K. mikimotoi* spacer sequence obtained by molecular cloning and is used as the target of PLP. The analysis of on-site samples shows that HRCA-LFD analysis is suitable for samples with target cell densities ranging from 1 to 1000 cells mL^−1^. HRCA-LFD can detect *K. mikimotoi* sensitively and reliably directly from seawater samples.

Najafzade has developed a method to accurately identify seven species of aquatic Exophiala species through RCA DNA PLPs [93]. The potential opportunistic species in the black yeast genus Exophiala are relatively high, and these opportunistic species can cause systemic or scattered infections in individuals with strong immune capabilities. Among them, the species that cause systemic diseases can generally grow at 37–40 °C, while other species lack heat tolerance, and most of them involve diseases of aquatic vertebrates, invertebrates, and most cold-blooded animals. Here, the author introduces a high-efficiency determination method that can identify and identify water-based Exophiala species without sequence restrictions. First, the author completed the sequencing and comparison of the ITS rDNA regions of seven Exophiala species and the closely related *Veronaea botryosa*. They designed a specific PLP that can be used to detect characteristic single nucleotide polymorphisms. By amplifying the DNA of the target fungus, the amplified product was observed on a 1% agarose gel to confirm the specificity of the probe-template binding and finally realize detection at the species level. During the experiment, the amount of reagents was reduced to prevent false-positive results. Simplicity, sensitivity, durability, and low cost make this PLP analysis (RCA) stand out in the diagnosis of bacterial DNA species. The application of terahertz (THz) spectroscopy in the field of sensing meets the needs of rapid and sensitive bacterial detection to a certain extent. Yang et al. developed an RCA-based THz biosensor for isothermal detection of bacterial DNA [94]. The first step is to hybridize a bacterial-specific, artificially synthesized 16S rDNA sequence with PLP, where the 5′ and 3′ ends of the PLP contain sequences that are completely complementary to the target sequence. When the target sequence is recognized and ligated, the linear PLP is circularized to form a circular PLP. Then, the capture probe (CP) immobilized on the magnetic beads plays the role of primer, and RCA starts to initialize. In the THz range, the absorbency of DNA molecules is far from that of water molecules, so the RCA product on the surface of the magnetic beads will cause the THz absorbance to decrease significantly. At this time, sensitive THz spectroscopy will detect the difference. The specificity test result is obvious, which is proven by its low signal response to interfering bacteria. The proposed strategy not only proves a new attempt to detect target bacterial DNA isothermally but also provides a general platform for sensitive and specific DNA biosensing using THz spectroscopy technology. Chen et al. used a research and development strategy that took full advantage of the sensitivity of HRCA to quickly detect *Amphidinium carterae* in environmental samples [97]. For coastal countries, the quality of marine water is decisive for the regional marine ecosystem, marine fisheries, or public health. Unfortunately, many coastal countries and regions are currently threatened by toxic microalgae, and they are becoming increasingly serious. Therefore, it is urgent and necessary to establish a large-scale water quality analysis and early warning system that can quickly, sensitively, and accurately detect toxin-producing microalgae in water bodies. In this article, the author uses HRCA to quickly detect *Amphidinium carterae*. First, the large subunit rDNA (LSU D1-D2) of *Amphidinium carterae* was sequenced to design a species-specific PLP. Then, the PLP combined with two amplification primers was used by HRCA. Of course, the HRCA sensing platform passed the specific detection experiment and passed the test with other algae. The entire operation process was controlled to be completed within 1.5 h. What is even more surprising is that the platform’s repeated detection limit is one cell. During the detection process, the fluorescent dye SYBR green I can be added to the amplified product so that the positive result of HRCA can be seen through the colour reaction. HRCA provides a very useful detection tool that can accurately screen large samples of *Amphidinium carterae* and other toxic species. To further improve the sensitive, Nie et al. applied HRCA and an HRCA-based strip test (HBST) for the detection of *Chattonella marina* [95]. As mentioned above, the existence of HAB poses a threat to marine ecosystems, fisheries and human health on a global scale. How to quickly and accurately monitor pathogenic algae and seawater quality is the goal. In this research article, the author combines the two methods and proposes the use of HRCA and HBST to rapidly detect *Chattonella* in the sea. The first is to sequence the large subunit (LSU) ribosomal DNA (rDNA) characteristic region of *Candida marinus* and design a specific PLP based on the sequencing results. In this way, the entire HRCA reaction covers two amplification primers and another HBST that plays an important role. The detection procedure involves a constant-temperature HRCA reaction, paper-based hybridization, and colour development results judged by the naked eye. Verifying specificity and sensitivity is an indispensable link. After a simple and logical operation, the results show that the detection limit of HBST detection is 1 copy μL^−1^ of the *Pseudomonas marina* LSU rDNA plasmid, which is the most prominent. It is one order of magnitude higher than the detection limit of HRCA and three orders of magnitude higher than the detection limit of conventional PCR. Finally, the author also applied the scheme to simulated field samples, and the results obtained are also good. The developed HBST still has higher detection sensitivity than HRCA and traditional PCR methods. In summary, the method proposed in this study is expected to break through the monitoring and early warning dilemma caused by HAB to the global ocean system and realize the on-site, sensitive and specific detection of cryptosporidium from natural samples. At the same time, it also provides good detection cases and models for future detection of other harmful algae.

Pearson et al. proposed the view that “virus recombination leads to fuzzy classification” [96]. The structural composition and dynamic changes of biological communities are inevitable research fields, especially in the application of agricultural sites. Research on microbial communities with high economic attributes and related maintenance is increasingly being discussed. The wastewater treatment plant is a melting pot with multiple coexistences. First, its sources are relatively wide, including not only domestic wastewater, livestock and poultry breeding wastewater but also various environmental wastewaters with stress factors, making wastewater treatment plants a variety of virus libraries of hosts that can be infected. In addition, the dynamic changes of its internal environment cannot be ignored; that is, the phenomenon of virus recombination cannot be ignored. They used a combination of sucrose gradient size selection and RCA to isolate the full-length genome of the circular ssDNA virus from the wastewater treatment facility and sequenced it on Illumina MSeq to achieve virus collection inspection. However, compared with the relatively large dsDNA viruses that are often studied, single-stranded DNA viruses are the least known microbial pathogens, and they also face technical bottlenecks such as genome bias and difficulty in cultivation. The team studied several typical examples, including model organisms (Microviridae) for genetic and evolutionary research and agricultural pathogens (Circoviridae and Geminiviridae) that infect livestock and crops. In the end, the results of the examination of viral DNA collected at the site provided evidence for 83 unique genotype groups. On the one hand, the results show the wide diversity of the community. These groupings are genetically different from known virus types; in addition, although the expression of these genomes is similar to that of known virus families, many differences are so great that they may represent the new taxonomy group. This study demonstrates the effectiveness of this method to isolate bacteria and large viruses from ssDNA viruses and the ability to use this protocol to obtain in-depth analysis of the diversity within the group.

Bejhed et al. used magneto-optical readings to realize simple, fast and cost-effective qualitative double-stranded detection of bacterial DNA sequences [98]. Whether it is the diagnosis of infectious diseases in biomedicine, the safety control of residents’ drinking water or food safety management and other health fields, the rapid, low-cost and easy-to-operate multitarget detection of pathogens has always been our unremitting pursuit. Therefore, we need an increasing number of novel bioassay methods to meet the requirements. Biological detection platforms established by magnetic and optical-magnetic bioassay methods are increasingly playing a role in developing countries due to their unique advantages. A photomagnetic method for the qualitative detection of bacterial DNA sequences has been developed, which can quickly determine whether the DNA sequence of the target bacteria appears in the sample. After two magnetic beads of different sizes are functionalized with nucleic acids, they hybridize with RCA products from two different bacteria to form the sensing platform. Among them, magnetic beads of different sizes are equipped with different oligonucleotide probes, which are only complementary to one of the RCA products. The final test results are also obtained from the same volume of samples. The measurement response is controlled by the modulation frequency of the applied magnetic field of the projected laser, which is an outstanding feature of the magneto-optical sensing platform. It is very suitable for countries and regions with low resources in the world, especially those that urgently need low-cost, large-scale screening for pathogens related to human or veterinary drugs.

In addition to the detection of microorganisms based on nucleic acids, RCA can also be used to detect living bacteria with aptamers. Ge et al. recently developed a method to detect *Salmonella typhimurium* directly [99]. The authors adopted an aptamer that hybridizes with the capture probe immobilized on the AuNP surface to recognize the target. In the presence of *Salmonella typhimurium*, the aptamer would combine with bacteria and release the primer binding site of the capture probe. This is followed by the triggering of the RCA reaction which produces various DNA fragments. Finally, the detection probe could be immobilized by the products of RCA via DNA hybridization, which could induce current change. This method has a very low detection limit of 16 CFU mL^−1^ and a broad linear range of 20 to 2 × 10^8^ CFU mL^−1^. This biosensor demonstrated that RCA has great potential for the direct detection of microorganisms with the help of aptamers.

## 4. Emerging Nanotechnology for RCA Assay

When constructing RCAs with higher performance analysis tools, in addition to new materials, several new uses of biotechnology, such as DNA integration technology and equipment, have also been developed. Among them, DNA technology has attracted much attention due to its programmed assembly and precise modelling of a strand-deforming operation. Moreover, RCAs can be used to construct various DNA machines to perform different functions via DNA technology. Additionally, engineering tools have also been widely used to make RCA more convenient and portable, such as microfluidics, paper devices and other commercial portable devices. When integrated with devices, RCA can be more easily used for point-of-care (POC) detection.

### 4.1. DNA Technology

#### 4.1.1. DNA Assembly Technology

DNA has been widely used to drive nanoparticle assembly due to its programmed assembly, high selectivity, and excellent recognition ability [136]. DNA assembly technology has also been applied to RCA assays, as it can generate a long ssDNA strand that can provide binding sites and various structures for assemblies [136].

Tian et al. recently constructed a biosensor to detect SARS-CoV-2 by combining RCA and DNA assembly technology [137]. The SARS-CoV-2 outbreak began in late December 2019 and soon spread around the world, which created a demand for rapid diagnosis [138]. The authors adopted a padlock probe (PLP) for target recognition followed by RCA for signal amplification and the assembly of MNPs for signal transduction. The padlock probe will be ligated to form circular templates for the first round RCA (CT1) when the target appears. Then, the intermediate amplicons will be generated by nicking-enhanced RCA. The intermediate amplicons could anneal to circular templates for the second round RCA (CT2) to generate amplicon coils, leading to the assembly of magnetic nanoparticles that could be detected by optomagnetic sensing. The method can be finished in 100 min with a dynamic detection range of three orders of magnitude and achieved a detection limit of only 0.4 fM. This method demonstrated that DNA assembly may provide good signal transduction for RCA detection.

RCA can also be used to construct organic polymer materials via DNA assembly technology such as DNA hydrogels. Na et al. used the RCA method and microbeads to generate a DNA hydrogel for the detection of infectious viruses [139]. The primer was first immobilized on the surface of microbeads, and the dumbbell-shaped templates were then immobilized via hybridization with primers. In the presence of the target nucleic acid, the templates can be circularized, and the RCA starts, which can generate a specific dumbbell shape to form the DNA hydrogel and block the flow path. Coloured ink was adopted as a visual indicator of whether the channel was blocked. The detection time was only 15 min, and the detection limit was 0.1 pM.

#### 4.1.2. DNA Machines

DNA machines refer to DNA molecules with several basic properties, such as the ability to perform mechanical functions accompanied by the need for fuel input, including pH and light, the generation of waste products, and energy consumption [140]. Recently, various DNA machines, such as tweezers, walkers, gears, and cranes, have been developed to perform different functions, such as drug delivery and control of the fluorescence properties of fluorophores [141,142].

**Figure 7:**
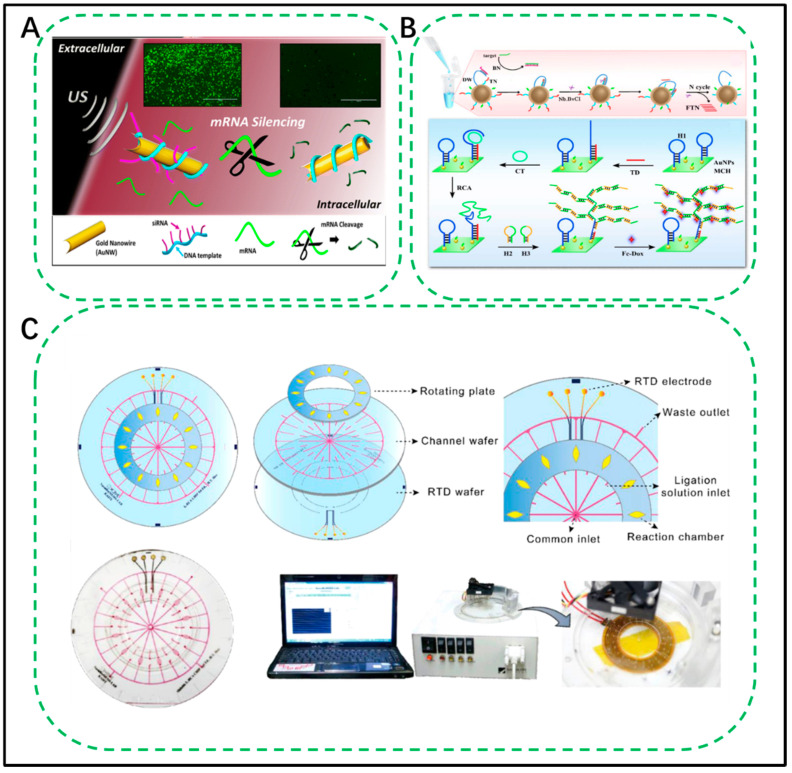
Emerging nanotechnology for RCA assay [141,143,145]. (**A**) Schematic of the nanomotor-based gene silencing approach; (**B**) Schematic representation of the multiple sensitizing electrochemical biosensor for the detection of *E. coli* O157:H7; (**C**) a novel rotary microfluidic device which can perform multiplex single nucleotide polymorphism typing on the mutation sites of TP53 genes.

An RCA assay could also be combined with a DNA machine, as it can generate DNA strands for the device. de Avila et al. designed acoustically propelled nanomotors for intracellular siRNA delivery by employing gold nanowires and the RCA method [141]. As shown in Figure 7A, the authors first utilized the RCA reaction to produce long ssDNAs made of GFP-targeting siRNA regions and noncoding spacer regions. Then, siRNA was bound to the long ssDNA strand and created alternating single-stranded and double-stranded RCA products, which could be wrapped on the positively charged surface of the gold nanomotor to form a DNA machine. This machine could be driven by ultrasound to penetrate the cell membrane. Once inside cells, the siRNA immobilized on the AuNW surface suppressed the gene-mRNA expression, making the cell fluorescence “OFF”. This DNA machine is an efficient RNA delivery tool and might be a promising platform for RNA-mediated gene therapy. The development of this method suggested that RCA could not only be applied to detection but also be a promising technique for gene therapy and drug delivery.

The DNA walker is another DNA machine that has been combined with the RCA assay. Li et al. recently developed a method for the detection of *Escherichia coli* O157:H7 based on an RCA assay and a DNA walker [143]. The method can be divided into three parts, as illustrated in Figure 7B. First, DNA walker-based amplification can be instigated by the presence of the target gene, which could hybridize with BN to release DW for further hybridization between DW and TN. DNA walker-based amplification could then be started with the help of Nb. BbvC I. The DNA generated by the DNA walker could open hairpin DNA 1 (H1) to activate the RCA reaction. The long ssDNA produced by RCA could react with hairpin DNA 2 (H2) and hairpin DNA 3 (H3) to start HCR amplification. Through the combination of the DNA walker, RCA and HCR, this biosensor possesses a detection limit of 7 CFU mL^−1^ and is superior to most detection assays for *E. coli* O157:H7.

### 4.2. Engineering of RCA as a Portable Tool for Point-of-Use Detection

Traditional detection methods such as ICP-MS and PCR are usually based on complex and expensive instruments. Instrumental assays have various advantages, including high sensitivity, stability, and selectivity. However, they usually require long and complex pretreatment steps, costly instruments, and professional operations. Therefore, they cannot be directly applied to POC detection. In recent years, several portable assays, such as microfluidic chips and paper devices, have been established for POC detection, and some of them can be combined with RCA assays.

#### 4.2.1. Microfluidic Chips

A microfluidic chip is an integrated platform that integrates microanalysis and pretreatment steps such as sampling, dilution, reagent addition and separation [144]. The platform possesses various advantages, including ease of control, low cost and low sample consumption, and is a promising technique for POC detection.

Heo et al. designed a valveless rotary microfluidic device based on the RCA assay that can detect multiple single-nucleotide polymorphisms simultaneously [145]. As shown in Figure 7C, the device consists of three components: a channel wafer, a resistance temperature detector (RTD) wafer with a Ti/Pt electrode pattern, and a rotating plate. The sample is loaded in the twelve ligation solution inlets, and the chamber of the top rotating plate is aligned with the radial microchannel. The padlock probe for recognition is also in the chamber. Then, the chamber was isolated by rotating the plate 7.5° for the ligation reaction and rotating again for RCA reagent injection. The RCA reaction and detection probe hybridization were conducted similarly to the ligation reaction. Finally, the results were read out via a fluorescence optical microscope. This microfluidic chip can not only achieve multiplex detection but also needs no microvalves or micropumps, simplifying the chip design and operation.

#### 4.2.2. Paper-Based Platforms

Paper materials that are abundant, low-cost, easy to manufacture, portable, and have support over sensor devices are widely used, especially in the POC diagnostic field. Liu et al. constructed a paper device for DNA or microRNA detection based on the RCA assay [146]. The authors first adopted the wax-printing technique to produce a 96-microzone paper plate with a test zone diameter of 4 mm. The RCA primer was also printed on the test zone. Then, the RCA reagents, including the circular DNA template, phi29 DNA polymerase, dNTPs and hemin, were mixed with pullulan solution and printed into the test zone. After air-drying, the paper device was finished. The addition of the target gene will activate the RCA reaction and can be detected by the colour change with TMB and H_2_O_2_ because its product possesses the PW17 sequence. This device successfully simplifies the detection steps and requires no expensive instrumentation. More importantly, it achieved comparable results to the values obtained using qRT-PCR.

#### 4.2.3. Electrochemistry Platforms

Electrochemical sensors have been widely used for point-of-care detection. Recently, the RCA method has also been applied for electrochemical sensors to detect pathogens, macromolecules, and small molecules. Huang et al. designed a biosensor based on RCA and voltametric methods to detect hepatitis B virus [147]. The method has an extraordinary sensitivity with 2.6 aM detection limit and the response is linear in the 10–700 aM range. Shen et al. established an immunoelectrochemical biosensor [148] based on the RCA method to detect human epidermal growth factor receptor 2 (HER2), and the detection limit was just 90 fg mL^−1^. Yi et al. recently developed a versatile electrochemical platform to detect adenosine with a detection limit of 320 pM and a linear range of 1 nM–10 μM [149]. In general, these methods are similar and utilize the specificity of RCA to recognize the target and the electrochemical effect of the RCA product (many could be indirect products, such as probes immobilized on the long ssDNA generated by the RCA reaction) to transduce the chemical signal into an electrical signal. Taking Yi’s study as an example, the conformation of the right probe was changed in the presence of adenosine, forming a hairpin structure. The hairpin structure can be linked to another hairpin structure generated by the left probe to produce circular DNA. Then, the RCA reaction can be triggered with primers and Phi29 polymerase [149]. The RCA product could hybridize with capture probes on the electrode surface which induced an increase in the impedance signal. This device is not only easy to operate but also flexible. Only primes need to be changed when they are used for the detection of different targets.

#### 4.2.4. Commercial Portable Device

RCA analysis is usually integrated with commercial small equipment for on-site analysis. Portable devices have rapid detection, ease of use, and low-cost characteristics and have been widely used in daily life.

The glucose metre might be one of the most successful commercial portable devices recently. Various efforts have been made to combine RCA methods with glucose metres. Jia et al. recently developed a biosensor to detect p53 DNA based on an RCA assay and glucose metre [150]. This paper first immobilized a hairpin probe on magnetic beads for recognition followed by a Padlock probe-mediated RCA step for signal amplification. Then, numerous DNA-invertase conjugations were tagged on the long ssDNA generated by the RCA assay to hydrolyse sucrose to glucose for detection by a glucose metre. This biosensor achieved a detection limit of 0.36 pM with a linear calibration range from 0.5 to 10 pM and exhibited excellent sequence selectivity. The above studies proved that RCA is a flexible assay method that can be combined with different platforms for application in different situations to satisfy different demands.

## 5. Conclusions and Perspective

The quality of the water environment is closely related to human health, food, energy and the economy. As mentioned earlier, RCA-based analysis technology is a reliable alternative for detecting various targets to track environmental pollutants. Due to its simplicity and high selectivity, different types of environmental pollutants can be monitored, and an increasing number of RCA-based analytical methods have been established. As a simple, efficient and temperature-free nucleic acid amplification tool, RCA has now become a powerful tool in the field of environmental monitoring. In particular, when RCAs and functional nucleic acids, including aptamers and DNA enzymes, as well as other assay platforms, PCR, ELISA, microfluidics, surface plasmon resonance (SPR), and nanoparticles, are integrated into ultrasensitive detection of various targets, including nucleic acids, proteins, small molecules, viruses, and cells. From the point of view of materials science, RCA project versatility makes it an exciting tool for the preparation of DNA building blocks and the construction of highly ordered nanostructures and new materials that may have practical applications in biosensing and environmental monitoring.

Furthermore, based on the synthesis of multivalent ligand binding variable RCA, many other multivalent ligand systems are difficult to achieve, including the number of ligands, density, type, and spatial organization, and they represent a new chemical biology tool in environmental monitoring. Despite the many advantages of the RCA system, there are still some challenges to overcome in practical applications. First, preparing a large number of high-purity circular templates may be an unavoidable challenge. For example, in enzymatic ligation methods, in addition to circular DNA, linear multimer byproducts can sometimes be formed. This problem can be partially solved by using low concentrations of DNA in the ligation reaction. Then, the circular DNA product can be purified from the linear byproduct by gel electrophoresis or exonuclease treatment, which only degrades noncircular linear DNA molecules. In addition, it is known that the enzymatic ligation process is effective for relatively large DNA substrates but may not be suitable for making small DNA loops (~30 nt). This may be due to an insufficient number of enzyme binding sites and/or is caused by the induced strain after restriction enzyme digestion. The closed loop of short oligonucleotides. However, this defect can be solved by chemically circularizing DNA oligonucleotides. This method can generate both small (~14 nt) and large circular templates (415 nt) with very good yields (up to 85%) of circular DNA molecules. In addition, other challenges in the practical application of RCA systems include mass production, purification, and storage because RCA products tend to aggregate for a long time due to non-specific intermolecular and intramolecular cross-linking. In addition, due to the large molecular weight of RCA products, nonspecific binding may occur when used in complex water environments such as wastewater. These problems can be minimized by fine-tuning the parameters, including RCA product length, order, composition, and stiffness. For instance, the authors found that the incorporation of polyT (rather than random sequence) spacers between the aptamer domains of RCA products can reduce nonspecific interactions between the multivalent aptamer system and various targets. Finally, computer-aided methods can be used to design RCA sequences and short chains to construct predictable DNA nanostructures to minimize unwanted nonspecific interactions. The analysis method based on RCA is an innovative approach for the simple and rapid detection of various targets in environmental monitoring and can even be applied to on-site detection. This method will open up a new direction for environmental pollution assessment, drug abuse trend assessment, public health assessment, and other fields.

## Figures and Tables

**Table 1:** Comparison of RCA, PCR and real-time PCR for the detection of DNA.

Features	Conventional PCR Assay	Real Time-PCR Assay	RCA Assay
Sensitivity	Sensitive	Highly sensitive	Highly sensitive
Specificity	Specific	Specific	Specific
Temperature conditions	Thermal cycle	Thermal cycle	Isothermal
Inhibition by biological samples	Yes	Yes	No
Instruments required	Thermocycler	Thermocycler	Not required
Post-assay analysis	Required	Required	Generally not required
Amplicon detection methods	Gel electrophoresis	Real-time detection/amplification graph	Gel electrophoresis, Turbidity measurement by visual inspection or using a real-time turbidimeter; dye-based visual detection
Qualitative detection	Yes	Yes	Yes
Quantitative detection	No	Yes	Semi-quantitative
Portability	Partially	Yes	Yes
Overall assay time	3–5 h	2.5–4 h	1–1.5 h
Cost effectiveness	Less expensive	Expensive	Less expensive

**Table 2:** Overview of RCA assays for the detection of targets in aqueous environments.

Targets		Detection Signal	Detection Range	LOD	Reference
Heavy metal ions	Hg^2+^	Fluorescence	0.42 pM–42.5 nM	0.14 pM	[74]
Heavy metal ions	Hg^2+^	Electrochemical	0.2 pM–100 nM	0.097 pM	[75]
Heavy metal ions	Hg^2+^	Fluorescence	0–20 nM	200 pM	[76]
Heavy metal ions	Hg^2+^	ECL	0.1 pM–0.1 μM	33 fM	[27]
Heavy metal ions	Hg^2+^	Electrochemical	1 pM–1 μM	0.684 pM	[77]
Heavy metal ions	Hg^2+^	Colorimetry	2.5–100 nM	1.6 nM	[78]
Heavy metal ions	Hg^2+^	Colorimetry	0–14 μg L^−1^	3.3 μg L^−1^	[79]
Heavy metal ions	Pb^2+^	Fluorescence	1.0–100 nM	1 nM	[80]
Heavy metal ions	Pb^2+^	pH values	1.0–100 nM	0.91 nM	[81]
Heavy metal ions	Pb^2+^	Fluorescence	0.1–50 nM	0.03 nM	[60]
Heavy metal ions	UO_2_ ^2+^	Colorimetry	0.02–15 ng mL^−1^	1.0 pg mL^−1^	[82]
Organic small molecules	Bisphenol A (BPA)	Fluorescence	1 nM–0.1 fM	5.4 × 10^−17^ M	[83]
Nucleic acids	miRNA	Fluorescence	50–500 fM	25 fM	[84]
Nucleic acids	miRNA	Fluorescence	10–10^6^fM	20 fM	[85]
Nucleic acids	R6G	Fluorescence	10^−16^–10^−11^M	8.7 × 10^−18^ M	[86]
Nucleic acids	gene point mutation	Fluorescence		1 μM	[87]
Peptides and proteins	microcystin-LR	Electrochemical	0.01–50 μg L^−1^	0.007 μg L^−1^	[88]
Peptides and proteins	glutamate dehydrogenase (GDH)	Fluorescence	10–100 nm	3 nM	[89]
Microorganisms	*Karenia mikimotoi*	Lateral flow assay	1–1000 cells mL^−1^	0.1 cell mL^−1^	[90]
Microorganisms	*Karenia mikimotoi*	Colorimetry	1–1000 cells mL^−1^	1 cell mL^−1^	[91]
Microorganisms	Harmful algal blooms (HABs)	Colorimetry	0.1–1000 cells mL^−1^	0.1 cell mL^−1^	[92]
Microorganisms	Exophiala	Electrophoresis	-	single-nucleotide level	[93]
Microorganisms	16S rDNA	THz absorption	10^−10^–10^−7^ M	0.6 × 10^−10^ M	[94]
Microorganisms	*Chattonella marina*	Fluorescence	10–10^5^ cells mL^−1^	10 cell mL^−1^	[95]
Microorganisms	circular ssDNA viruses	Whole-genome sequencing	-		[96]
Microorganisms	*Amphidinium carterae*	Electrophoresis	100 ng mL^−1^–1 fg mL^−1^	281 copies	[97]
Microorganisms	bacterial DNA sequences	Optical (laser)	-	one bacterial DNA sequence	[98]
Living bacteria	Salmonella typhimurium	Current	20–2 × 10^8^ CFU mL^−1^	16 CFU mL^−1^	[99]
Other targets	ATP	Droplet motion	50 pM–5 mM	5 nM	[100]

## Data Availability

No new data were created or analyzed in this study. Data sharing is not applicable to this article.
